# Frailty and the risk of dementia: is the association explained by shared environmental and genetic factors?

**DOI:** 10.1186/s12916-021-02104-3

**Published:** 2021-10-18

**Authors:** Ge Bai, Yunzhang Wang, Ralf Kuja-Halkola, Xia Li, Yasutake Tomata, Ida K. Karlsson, Nancy L. Pedersen, Sara Hägg, Juulia Jylhävä

**Affiliations:** 1 Department of Medical Epidemiology and Biostatistics, Karolinska Institutet, Nobels väg 12A, 17165 Stockholm, Sweden; 2 School of Nutrition and Dietetics, Faculty of Health and Social Services, Kanagawa University of Human Services, Yokosuka, Japan; 3 Institute of Gerontology and Aging Research Network – Jönköping (ARN-J), School of Health and Welfare, Jönköping University, Jönköping, Sweden; 4 Faculty of Social Sciences (Health Sciences) and Gerontology Research Center (GEREC), University of Tampere, Tampere, Finland

**Keywords:** Frailty, Dementia, Twin design, Cohort study, Genetic factors

## Abstract

**Background:**

Frailty has been identified as a risk factor for cognitive impairment and dementia. However, it is not known whether familial factors, such as genetics and shared environmental factors, underlie this association. We analyzed the association between frailty and the risk of dementia in a large twin cohort and examined the role of familial factors in the association.

**Methods:**

The Rockwood frailty index (FI) based on 44 health deficits was used to assess frailty. The population-level association between FI and the risk of all-cause dementia was analyzed in 41,550 participants of the Screening Across the Lifespan Twin (SALT) study (full sample, aged 41–97 years at baseline), using Cox and competing risk models. A subsample of 10,487 SALT participants aged 65 and older who received a cognitive assessment (cognitive sample) was used in a sensitivity analysis to assess the effect of baseline cognitive level on the FI-dementia association. To analyze the influence of familial effects on the FI-dementia association, a within-pair analysis was performed. The within-pair model was also used to assess whether the risk conferred by frailty varies by age at FI assessment.

**Results:**

A total of 3183 individuals were diagnosed with dementia during the 19-year follow-up. A 10% increase in FI was associated with an increased risk of dementia (hazard ratio [HR] 1.17 (95% confidence interval [CI] 1.07, 1.18)) in the full sample adjusted for age, sex, education, and tobacco use. A significant association was likewise found in the cognitive sample, with an HR of 1.13 (95% CI 1.09, 1.20), adjusted for age, sex, and cognitive level at baseline. The associations were not attenuated when adjusted for *APOE* ɛ4 carrier status or considering the competing risk of death. After adjusting for familial effects, we found no evidence for statistically significant attenuation of the effect. The risk conferred by higher FI on dementia was constant after age 50 until very old age.

**Conclusions:**

A higher level of frailty predicts the risk of dementia and the association appears independent of familial factors. Targeting frailty might thus contribute to preventing or delaying dementia.

**Supplementary Information:**

The online version contains supplementary material available at 10.1186/s12916-021-02104-3.

## Background

A systematic analysis for the Global Burden of Diseases, Injuries, and Risk Factors Study 2016 reported that the number of people worldwide living with dementia has more than doubled from 1990 to 2016 [1]. According to Swedish register data, there are an estimated 150,000 dementia patients and approximately 24,000 individuals develop dementia each year [2]. Lower education, hypertension, hearing impairment, smoking, obesity, depression, physical inactivity, diabetes, low social contact, excessive alcohol consumption, traumatic brain injury, and air pollution were recently identified in the 2020 report of the Lancet Commission [3] as 12 common risk factors for incident dementia. However, because current medical treatment cannot cure or reverse dementia, but only alleviate the symptoms, identifying new potential modifiable risk factors is crucial in preventing or delaying the onset of dementia.

Meta-analyses and systematic reviews have reported that frailty predicts incident dementia [4, 5]. The studies have, however, almost exclusively focused on individuals aged 65 and older, leaving younger and middle-aged adults understudied. The association appears robust regardless of the scale used to measure frailty; both the frailty phenotype (FP) [6] and frailty index (FI) [7] are predictive of dementia. However, the mechanisms underlying the association are unclear. Frailty and dementia share several risk factors and clinical manifestations [8] and may even share a common pathological basis [9]. It is also possible that some genetic variants increase the risk of both conditions or modify the effect of environmental and lifestyle-related factors, such as education, smoking, and physical activity, on frailty and dementia. Twin studies have shown that Alzheimer’s disease, the most common form of dementia, is highly heritable [10], with the *apolipoprotein E* (*APOE*) ɛ4 allele being the strongest genetic risk factor [11]. Although the genetics of frailty are less well understood, twin studies have estimated that the heritability of the FI ranges from 30 to 52% [12, 13], and a genome-wide association study has indicated that variants in brain pathways underlie the risk of frailty [14].

In this study, we sought to address the gaps in current evidence by analyzing whether frailty predicts dementia in a large cohort of twins including younger and older adults and discerning whether the association is explained by genetic and/or shared environmental factors. As monozygotic (MZ) twins can be considered genetically identical, whereas dizygotic (DZ) twins share on average 50% of their segregating genes, associations within twin pairs discordant for dementia can inform about the involvement of genetic and shared environmental factors [15]. Should there be no involvement (“confounding”) of genetic and shared environmental factors, the effect size of the exposure (frailty) observed in the population-level analysis would have to persist in the within-pair analysis in both DZ and MZ twins. Such a scenario would be consistent of at least partially causal relationship between frailty and dementia, making frailty a potential target for the prevention of dementia. A similar attenuation of the effect in both DZ and MZ twins would indicate the involvement of shared environmental factors, including but not limited to early life exposures and lifestyle-related factors. The familial environmental factors are thus all types of “anonymous” influences that make the twins similar to each other—even in later life. Further attenuation of the effect in MZ twins relative to DZ twins would indicate that shared genetic influences explain the association between frailty and dementia as genetic effects are fully accounted for in the within-MZ pair analysis. In such a scenario, intervening the exposure (decreasing frailty) is unlikely to lead in the effective improvement of the outcome (preventing dementia).

## Methods

### Study population

The data came from the Screening Across the Lifespan Twin Study (SALT) [16, 17] which was conducted in 1998–2002 on all then living twin individuals born in 1958 or earlier (aged between 41 and 97 years) who were included in the Swedish Twin Registry [18]. SALT collected data on diseases, symptoms, lifestyle factors, and medication use through a computer-assisted telephone interview.

**Fig. 1:**
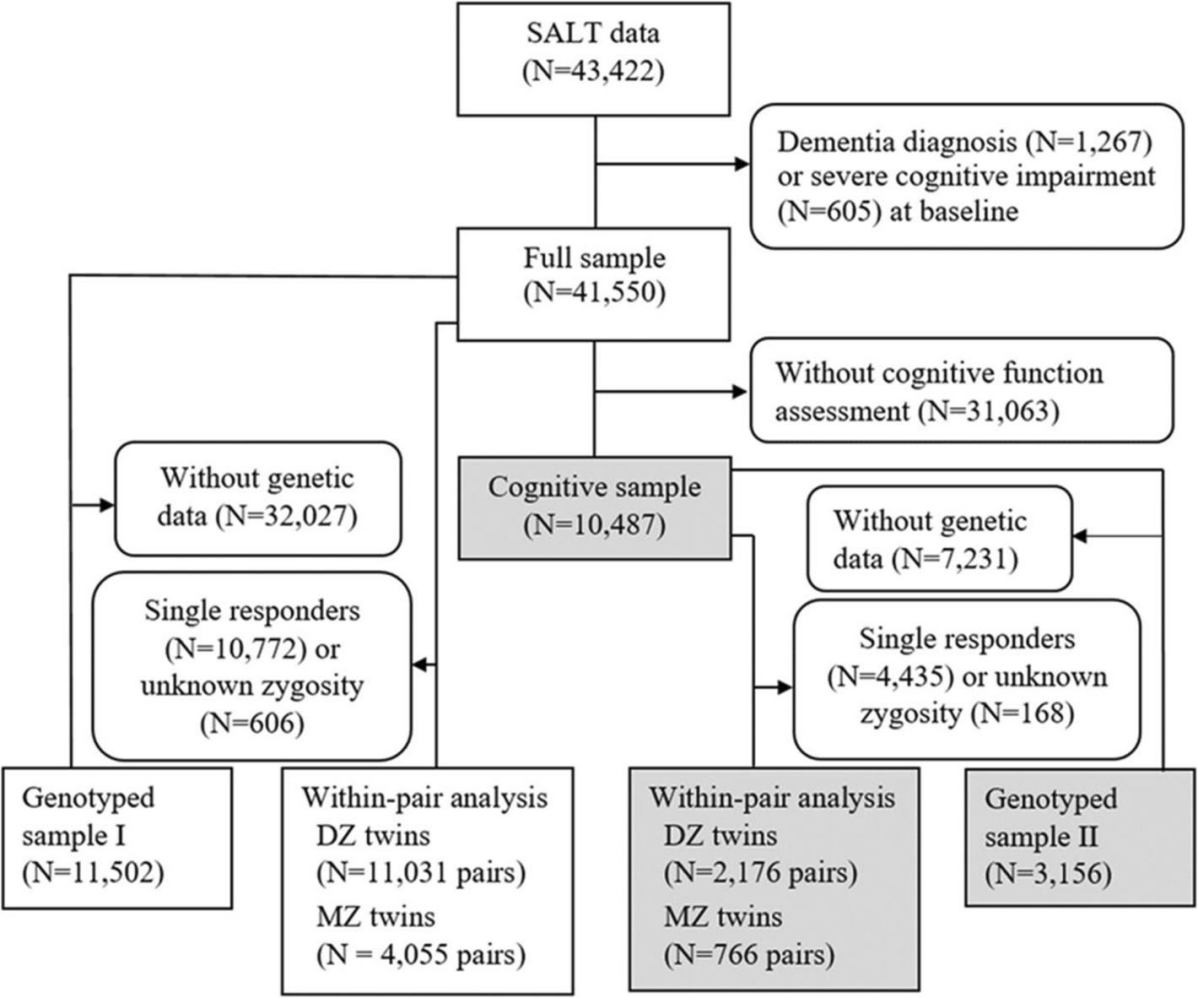
Flow chart of the study participants. The cognitive sample (indicated in gray) was used for sensitivity analysis (results presented in Additional file 1). Dizygotic (DZ) and monozygotic (MZ) twin pairs were included in the within-pair analysis

The selection of the study population is shown in Fig. 1. After linking to national health register data on dementia diagnoses using personal identification number, we excluded individuals with an onset of dementia before baseline or who had severe cognitive impairment at baseline (see below). This left us with 41,550 participants (full sample) available for the main analysis. A subsample of 10,487 individuals in SALT aged 65 years and older who received a cognitive assessment at baseline (cognitive sample) was used in a sensitivity analysis and to further adjust for baseline cognitive level. Given the significant role of the *APOE* ɛ4 allele as a risk factor of dementia, we further performed analyses adjusting for *APOE* ɛ4 carrier status in individuals with genotype data available. A subsample of 10,502 participants from the full sample (genotyped sample I) and a subsample of 3156 participants in the cognitive sample (genotyped sample II) were available for this analysis. Lastly, for the within-pair analysis, we had 11,031 DZ twin pairs and 4055 MZ twin pairs available in the full sample and 2176 DZ twin pairs and 766 MZ twin pairs in the cognitive sample (Fig. 1).

### Assessment of the FI

Construction of the FI used in this study has been described previously [19]. Briefly, the FI is based on the Rockwood deficit accumulation model [7] and consists of 44 self-reported health-related items on general health status, diseases, signs and symptoms of disease, psychosocial health, and functional abilities. We included all available items in SALT that meet the standard inclusion criteria [7] and are also suitable for younger adults (< 65 years). For example, activities of daily living were only available for those aged 65 and older and were thus not included. The missing values across the FI items were imputed by chained equations as previously described [19]. A list of the included FI items and their coding is presented in Additional file 1: Table S1 [20–22]. The FI for each individual was calculated as the number of deficits present divided by the total number of deficits, yielding a continuous score ranging from 0 to a theoretical maximum of 1.

### Ascertainment of all-cause dementia

Dementia diagnoses during the follow-up were retrieved from nationwide registers, namely, the National Patient Register (NPR), the Cause of Death Register (CDR), and the Prescribed Drug Register (PDR) [23]. Both the NPR (with nationwide coverage since 1987) and the CDR (with nationwide coverage since 1961) contain disease information based on the International Classification of Diseases (ICD) system. Dispensed dementia medication according to Anatomical Therapeutic Chemical (ATC) codes in the PDR was regarded as proxy for dementia diagnosis. ATC codes for anti-dementia drugs in the N06D group were considered. The primary dementia diagnosis or death was followed up from baseline until the last day of December in 2016, yielding an up to 19 years of follow-up. The ICD and ATC codes used to identify dementia are shown in Additional file 1: Tables S2 and S3. In addition, dementia information was available from ascertainments from STR studies of aging, including SALT for participants aged 65 or older (the cognitive sample), where diagnoses were set at consensus conferences based on DSM-III-R and DSM-IV criteria [20].

### Covariates

In addition to sex and age at FI measurement, body mass index (BMI), tobacco use, years of education, living alone, and physical activity were considered as covariates because of their roles as common risk factors for dementia [3]. BMI, from self-reported data, was calculated as weight divided by height squared (kg/m^2^). Tobacco use was classified as non-user (reference category) or user if the participant was a current smoker or used smokeless tobacco regularly, had previously smoked, or used smokeless tobacco regularly. Living alone was a binary variable with “not living alone” as the reference category. For those who were born after 1926 (*N* = 37,218), physical activity was assessed based on the question “Of these 7 alternatives, which fits your annual exercise pattern?” The alternatives were 0 (almost never exercise), 1 (much less exercise than average), 2 (less exercise than average), 3 (average amount of exercise), 4 (more exercise than average), 5 (much more exercise than average), and 6 (maximum amount of exercise). For those who were born before 1926 (*N* = 4046), physical activity was assessed based on the question: “How much do you exercise?” The alternatives were 0 (almost no exercise), 1 (light exercise), 2 (regular median exercise), and 3 (hard physical exercise). The two physical activity variables were transformed to *z* scores (each unit representing one standard deviation from the mean) and combined into one variable for the analysis. Screening for cognitive function for those aged 65 and older (cognitive sample) is described in Additional file 1, Appendix S1: Supplementary methods.

#### Genotype data

*APOE* ɛ4 carrier status was additionally adjusted for in the genotyped sample I (*N* = 10,502) and genotyped sample II (*N* = 3156). The *APOE* ɛ4 genotypes were either directly genotyped or determined from Illumina OmniExpress imputed to 1000 Genomes Project [24] using a pipeline with high accuracy [25]. Individuals carrying the genotypes ɛ2/ɛ2, ɛ2/ɛ3, or ɛ3/ɛ3 were categorized as non-carriers (reference category); those carrying the genotype ɛ2/ɛ4 or ɛ3/ɛ4 were categorized as heterozygous; and those carrying the genotype ɛ4/ɛ4 were categorized as homozygous.

### Statistical analysis

Two main analytical approaches were used in this study: a cohort analysis to obtain population-level estimates of the association between frailty and dementia and a within-pair analysis to control for familial (i.e., genetic and shared environmental) effects on the association. All analyses were performed in the same manner in the full and cognitive samples.

We first performed Cox proportional hazard models with time since the FI measurement as the underlying timescale to estimate the hazard ratios (HRs) for a 10% increase (i.e., 0.1 increment) in the FI on dementia. Individuals who died during the follow-up were censored at the date of death. Age at FI assessment, sex, BMI, tobacco use, years of education, living alone, physical activity, and cognitive function score (in the cognitive sample, described in Additional file 1, Appendix S1: Supplementary methods) were first tested for their association with dementia in univariate Cox models. Following the rule of parsimony, those variables that were statistically significant or had an effect on the FI estimate were included as covariates in the multivariate Cox model. The proportional hazard assumption was tested using an interaction term between the covariates and time in the model. *APOE* ɛ4 carrier status was additionally adjusted for in the multivariate Cox models in the genotyped samples I and II that consisted of individuals who had genotype data available (Fig. 1). Cluster-robust standard errors were used to correct for the correlation within twin pairs. Next, taking into account the competing risk of death, a competing risk model based on the Fine and Gray method using subdistribution HRs (SHRs) [26] was performed in the full and cognitive samples. The functional form of the relationship between the FI and dementia was assessed using the log-likelihood test between the quadratic and the linear model and plotting the log-HRs for a quadratic, cubic spline-transformed and linear FI. Lastly, to facilitate clinical interpretations, we assessed the relationship between categorized FI and the incidence of dementia by categorizing the FI into three levels: non-frail (FI ≤ 0.08), pre-frail (0.08 < FI ≤ 0.25), and frail (FI > 0.25), according to pre-established cut-offs [27]. The categorized FI (non-frail as reference) was then tested for its association with dementia in a Cox model. Kaplan-Meier curves were used to assess the probability of being dementia-free during the follow-up by the aforementioned frailty categories in the full and cognitive samples.

The within-pair analysis was conducted in DZ and MZ twin pairs that were complete, i.e., both members of the pair had relevant information on FI and dementia (Fig. 1). A between-within (BW) model, a random-effects model incorporating a BW decomposition [22], was applied in a survival analysis framework to conduct the within-pair analysis. In this analysis, we tested the time-constant within-pair effect of the FI on dementia. To explore whether the associations differed by sex, the within-pair analysis was additionally performed separately in men and women (like sexed twin pairs). To facilitate conclusions about the independent, potentially causal role of frailty on dementia, we used a Wald test to formally test whether the within-pair estimate in MZ pairs differed from the population-level estimate in the Cox model for the MZ twins. We fitted the two models in parallel to allow for the statistical test of the two regression coefficients.

To assess whether the risk carried by increased FI varies over age at FI measurement and whether the association is independent of familial effects throughout the age range, from adulthood into old age, we modeled the association between FI and dementia including a statistical interaction between FI and age and modeled this interaction as a natural cubic spline function. We fitted the model to the standard association (population-level estimate), and to the within-pair association, henceforth referred to as the “standard interaction model” and the “within-pair interaction model,” respectively. To further test for genetic effects in the association between frailty and dementia, we formally assessed the difference in the MZ and DZ estimates across the age range in the within-pair interaction model by deriving the ratio of the HR between DZ and MZ (HR_DZ_/HR_MZ_) as a function of age. Details of the models are presented in Additional file 1, Appendix S1: Supplementary methods.

Lastly, as our FI included a number of conditions that are known risk factors for dementia, similar to the study by Song et al. [28], we conducted a sensitivity analysis by dividing the FI into those items that are traditional risk factors for dementia (FI-TRF) and those that are not (FI-NTRF) and analyzed them separately using Cox regression. Hypertension, high cholesterol or triglycerides, cerebral hemorrhage or stroke, TIA attacks, irregular cardiac rhythm/atrial fibrillation, diabetes, kidney disease, migraine, depression, and hearing were included in the FI-TRF due to their established roles as dementia risks [3, 29–31] and the remaining 34 items in the FI-NTRF (Additional file 1: Table S1). A two-sided *P*-value < 0.05 was considered statistically significant. All analyses were performed using STATA 15.1 and R version 3.6.1.

## Results

### Descriptive statistics

Characteristics of the study sample are presented in Table 1. Of the 41,550 individuals in the full sample (mean age = 58.0, SD = 10.1), 3183 were diagnosed with dementia, 7940 died, and 30,427 were censored during the up to 19-year follow-up. Descriptive statistics for the cognitive sample are presented in Additional file 1: Supplementary results and Table S4. The incidence rate of dementia was similar in DZ and MZ twin individuals in the within-pair sample I (Table 1). Characteristics of the analytical samples by dementia status and sex are presented in Additional file 1: Tables S5 and S6, respectively. The FI distribution was skewed with a long right tail (Additional file 1: Figure S1).

**Table 1:** Descriptive statistics of the full sample and the within-pair sample I. Data presented for the dizygotic (DZ) and monozygotic (MZ) twins includes those individuals who were available for the within-pair analysis. Values are mean (standard deviation, SD) unless otherwise indicated

	Full sample	Within-pair sample I	
	*N* = 41,550	DZ twin individuals *N* = 22,062	MZ twin individuals *N* = 8110
Age at baseline	58.0 (10.1)	56.7 (9.1)	56.6 (9.1)
Age range at baseline	41–97	41–91	41–88
Women, *N* (%)	22,193 (53.4)	11,621 (52.7)	4606 (56.8)
BMI	25.0 (3.5)	25.0 (3.5)	24.9 (3.5)
Tobacco user, *N* (%)	24,491 (58.9)	13,282 (60.2)	4549 (56.1)
Years of education	10.6 (3.2)	10.7 (3.2)	11.0 (3.2)
^§^Physical activity, median (IQR)			
Born before 1926	1 (1)	1 (1)	1 (1)
Born after 1926	3 (2)	3 (2)	3 (2)
Living alone, *N* (%)	9005 (21.7)	4395 (19.9)	1558 (19.2)
FI, median (IQR)	0.108 (0.108)	0.102 (0.108)	0.102 (0.108)
Categorized FI			
Non-frail, *N* (%)	15,464 (37.2)	8557 (38.8)	3133 (38.6)
Pre-frail, *N* (%)	22,354 (53.8)	11,757 (53.3)	4298 (53.0)
Frail, *N* (%)	3732 (9.0)	1748 (7.9)	679 (8.4)
Dementia diagnosis during follow-up, *N* (%)	3183 (7.7)	1364 (6.2)	494 (6.1)
Time to diagnosis, median (IQR)	16.0 (2.4)	16.1 (2.3)	16.1 (2.2)
Died during follow-up, *N* (%)	9932 (23.9)	2012 (9.1)	756 (9.3)

Note. Participants who used tobacco products include current smokers, ex-smokers, and snuff users at baseline

^§^Physical activity was assessed using a different questionnaire for those born before 1926 vs after 1926

*Abbreviations*: *BMI* body mass index, *DZ* dizygotic, *FI* frailty index, *IQR* interquartile range, *MZ* monozygotic, *N* number

### FI-dementia association at the population level

Assessing the functional form of the association between the FI and the risk of dementia in the full sample model indicated that there was not a statistically significant difference (*P* = 0.07) between a linear and quadratic fit. Further plotting of the log-HRs for the quadratic, cubic spline-transformed and linear FIs yielded similar estimates in the FI range 0–0.4 (Additional file 1: Figure S2) where ~ 98% individuals in SALT have their FI (Additional file 1: Figure S1). Hence, a linear approximation was used in the analysis. Age at FI assessment, sex, tobacco use, and years of education were statistically significantly associated with dementia in the multivariate Cox model and hence included as covariates. The assumption of proportional hazards was met for the FI. In the full sample, the univariate (adjusted for age and sex) and the multivariate Cox models showed that a 10% higher FI (i.e., increment of 0.1) was associated with a 19% (HR 1.19; 95% CI 1.14, 1.24) and 17% (HR 1.17; 95% CI 1.13, 1.23) increase in the risk of incident dementia, respectively (Table 2). We next performed analyses adjusting for *APOE* carrier status in the genotyped sample I, which consisted of individuals with genotype data available in the full sample. As the sample size decreased from the main analytical samples, to facilitate comparison, we first performed the multivariate Cox analysis in the genotyped sample I without adjusting for *APOE* ɛ4 carrier status (Table 2, model 1 in the right panel) and then adjusted for it (Table 2, model 2 in the right panel). The effect size of the FI remained unchanged when adjusting for the *APOE* ɛ4 carrier status (Table 2 right panel, model 1 vs. model 2). The corresponding models for the cognitive sample are presented in Additional file 1, Appendix S2: Supplementary results and Table S7. When considering the competing risk of death, the FI was statistically significantly associated with the risk of dementia risk in both univariate (adjusted for age and sex) and multivariate competing risk models (Additional file 1: Table S8).

**Table 2:** Association of the frailty index (FI) with the risk of dementia in the full sample (left panel) and in the genotyped sample I adjusting for the *APOE* ɛ4 carrier status (right panel). Hazard ratios (HRs) from the Cox regression and 95% confidence intervals (CIs) are presented for a 10% increase in FI

	Multivariate Cox models		Multivariate Cox models adjusting for the ***APOE*** ɛ4 carrier status	
	Full sample (*N* = 41,550)		Genotyped sample I (*N* = 11,502)	
	Model 1	Model 2	Model 1	Model 2
	HR (95% CI)	HR (95% CI)	HR (95% CI)	HR (95% CI)
FI	1.19 (1.14, 1.24)*	1.17 (1.13, 1.23)*	1.13 (1.04, 1.23)*	1.13 (1.03, 1.23)*
Age at FI measurement	1.15 (1.14, 1.16)*	1.15 (1.14, 1.16)*	1.15 (1.14, 1.16)*	1.16 (1.15, 1.17)*
Male sex	0.85 (0.78, 0.91)*	0.87 (0.80, 0.94)*	0.83 (0.72, 0.97)	0.82 (0.71, 0.96)*
Education years		0.97 (0.96, 0.98)*	0.98 (0.96, 1.00)	0.98 (0.96, 1.00)
Tobacco user		1.19 (1.10, 1.29)*	1.17 (1.01,1.35)*	1.16 (1.00, 1.34)*
*APOE* ɛ4 status (ref. non-carrier)				
Heterozygous (ɛ2/ɛ4 or ɛ3/ɛ4)				2.04 (1.75, 2.37)*
Homozygous (ɛ4/ɛ4)				7.02 (5.21, 9.46)*

Note. Model 1 in each sample adjusts for age and sex and model 2 adjusts additionally for education and tobacco use. Model 1 for the genotyped sample represents the FI-dementia association in this sample without adjusting for the *APOE* ɛ4 status and model 2 adjusts for the *APOE* ɛ4 status. *P < 0.05

The Kaplan-Meier curve for the probability of being dementia-free during the follow-up showed that frail and pre-frail individuals had a higher risk of developing dementia than non-frail individuals in both the full and cognitive samples, with a pattern that suggests a dose-response relationship between the FI categories (non-frail, pre-frail, and frail) and dementia (Additional file 1: Figure S3). A Cox regression for the categorized FI and the risk of dementia is shown in Additional file 1: Table S9.

### Within-pair analysis

Although we assessed both within- and between-pair effects in the BW model, we only present the within-pair effects for the FI and the covariates. The BW model outputs are henceforth referred to as within-pair models or within-pair analyses. As the sample size in the within-pair analysis decreased from the main analytical sample (the full sample), we first fitted multivariate Cox models in within-sample I for DZ and MZ twins (Table 3, left 2 panels). In the within-pair models, the within-pair effect of the FI in DZ twin pairs remained unchanged compared to the corresponding effect in the multivariate Cox model (Table 3, DZ twins left vs. right panel). The effect size of the MZ twins likewise remained unchanged in the within-pair model (Table 3, MZ twins left vs. right panel), yet the significance was attenuated. Formal testing of the difference in the population-level estimate in the Cox regression and within-pair estimate in the within-pair model for MZ twins (Table 3) revealed no significant difference in the estimates (*P* = 0.82). The within-pair analysis for the cognitive sample is presented in Additional file 1, Appendix S2: Supplementary results and Table S10.

**Table 3:** Association of the frailty index (FI) with the risk of dementia in complete DZ and MZ twin pairs in the within-pair sample in the multivariate Cox model (left panel) and the within-pair model (right panel). Hazard ratios (HRs) and 95% confidence intervals (CIs) are presented for a 10% increase in FI

	Within-pair sample I			
	Multivariate Cox model		Within-pair model	
	DZ twins *N* = 11,031 pairs	MZ twins *N* = 4055 pairs	DZ twins *N* = 11,031 pairs	MZ twins *N* = 4055 pairs
	HR (95% CI)	HR (95% CI)	HR (95% CI)	HR (95% CI)
FI	1.23 (1.15, 1.31)*	1.12 (1.00, 1.25)*	1.24 (1.12, 1.37)*	1.13 (0.91, 1.42)
Age at FI measurement	1.15 (1.14, 1.16)*	1.14 (1.12, 1.15)*	1.17 (1.16, 1.18)*	1.18 (1.16, 1.20)*
Male sex	0.83 (0.74, 0.94)*	0.87 (0.70, 1.08)	0.77 (0.65, 0.92)*	0.89 (0.68, 1.17)
Education years	0.97 (0.95, 0.99)*	0.96 (0.93, 0.99)*	0.97 (0.93, 1.00)	0.97 (0.89, 1.04)
Tobacco user	1.13 (1.00, 1.27)*	1.28 (1.04, 1.57)*	1.17 (0.96, 1.41)	0.78 (0.51, 1.18)

*P < 0.05

In the sex-stratified models for DZ and MZ twin pairs, there was no significant decrease in the within-pair effect size compared to the multivariate Cox in women (Additional file 1: Table S11). The same was observed for DZ men, whereas the effect of the FI in MZ men was attenuated relative to the DZ estimate, in both the Cox and within-pair models (Additional file 1: Table S11).

**Fig. 2:**
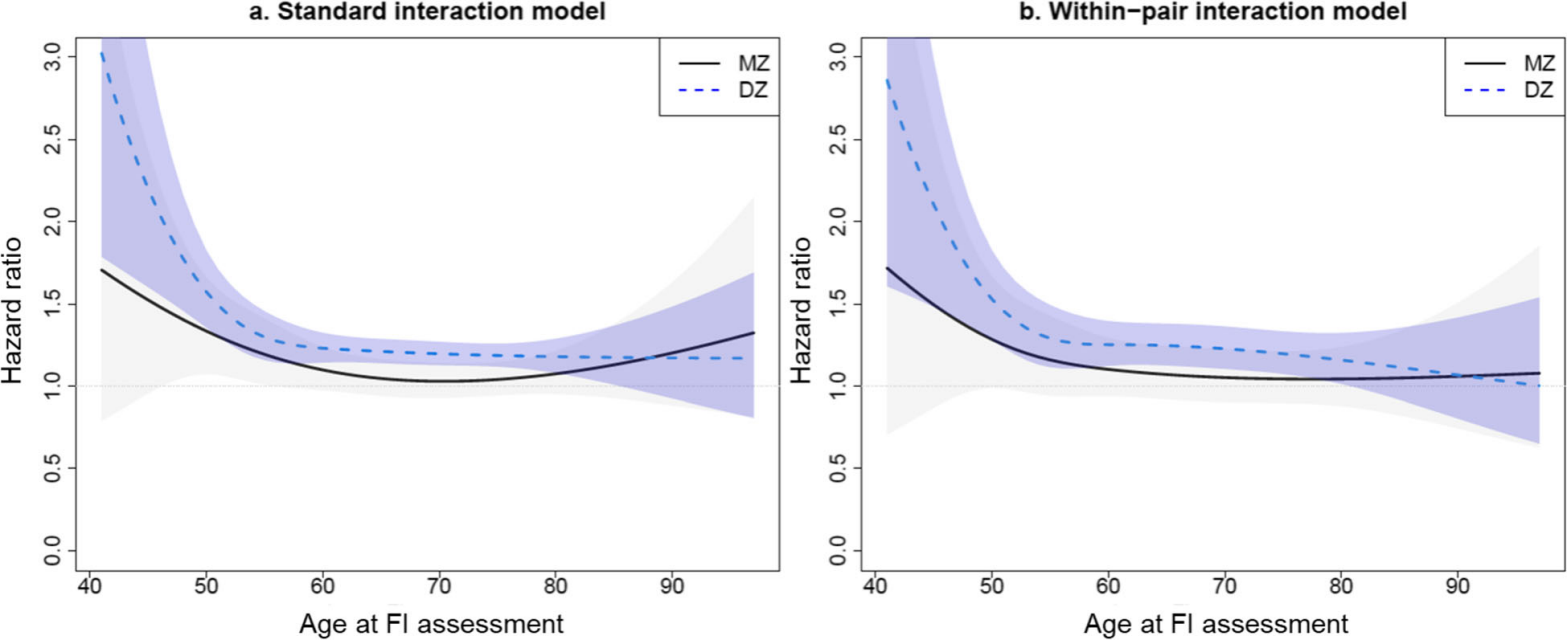
Age-varying effects of the frailty index (FI) on dementia in dizygotic (DZ) and monozygotic (MZ) twin pairs in the within-pair sample I. Age refers to the age at FI assessment. The standard interaction model (**a**) represents the Cox model adjusted for sex, age at FI measurement, years of education, tobacco use, and interaction terms between FI and zygosity and FI and age at FI measurement. The within-pair interaction model (**b**) additionally controls for familial factors. The dashed line represents the age-varying estimates in DZ twins and the solid line in MZ twins

Plotting the effect of the FI over age at FI assessment separately in MZ and DZ twins illustrated a similar pattern of risk across age at FI assessment in both the standard interaction and within-pair interaction models (Fig. 2). Both models were adjusted for sex, years of education, and tobacco use. For the DZ twins, but not for MZ twins, there was a significant decrease in the effect size between ages 40 and 50, after which the risk was seemingly constant across age. Although the DZ estimate appeared higher than the MZ estimate for most of the age range, the effect sizes did not differ significantly between MZ and DZ pairs, as indicated by the HR_DZ_/HR_MZ_ (varying over age at FI assessment) in the within-pair interaction model (Additional file 1: Figure S4).

### Sensitivity analysis with the FI-TRF and FI-NTRF

**Fig. 3:**
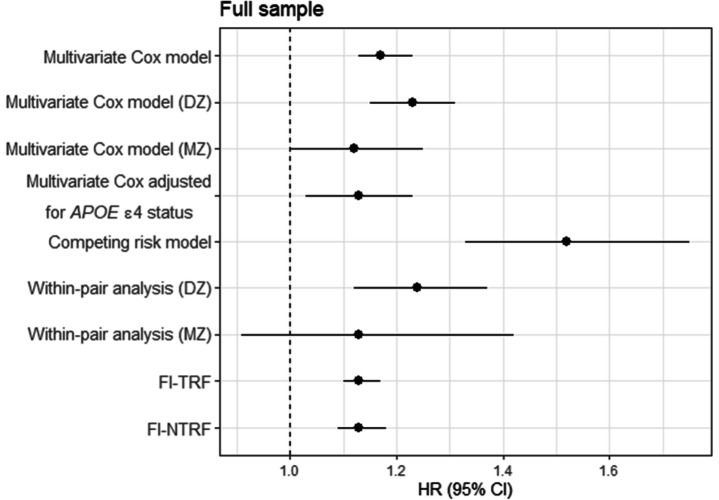
Hazard ratios (HRs) and the subdistribution HR (SHR) for the competing risk model and 95% confidence intervals (CIs) of incident dementia in relation to a 10% increase in the frailty index in the full sample. Abbreviations: FI-NTRF, frailty index constructed from non-traditional dementia risk factors; FI-TRF; frailty index constructed from traditional dementia risk factors; DZ, dizygotic; MZ, monozygotic

Both the FI-TRF and FI-NTRF were significantly associated with dementia in Cox regression models in the full sample (Table 4) and cognitive sample (Additional file 1: Table S12) after adjusting for age, sex, education, tobacco use, and cognitive level (in the cognitive sample). To facilitate comparison, all estimates from the multivariate Cox models, competing risk modes, and within-pair models are presented in Fig. 3 and Additional file 1: Figure S5.

**Table 4:** The associations between dementia and the frailty index (FI) constructed from traditional risk factors for dementia (FI-TRF, model 1) and non-traditional risk factors for dementia (FI-NTRF, model 2) assessed by Cox regression in the full sample adjusted for age, sex, education, and tobacco use. Hazard ratios (HRs) and 95% confidence intervals are presented for a 10% increase in the FI-TRF and FI-NTRF

	Full sample	
	Model 1 HR (95% CI)	Model 2 HR (95% CI)
FI-TRF	1.13 (1.10, 1.17)*	
FI-NTRF		1.13 (1.09, 1.18)*
Male sex	0.90 (0.83, 0.98)*	0.87 (0.80, 0.94)*
Age at FI measurement	1.15 (1.14, 1.16)*	1.15 (1.14, 1.16)*
Years of education	0.97 (0.96, 0.98)*	0.97 (0.96, 0.98)*
Tobacco user	1.21 (1.11, 1.30)*	1.19 (1.10, 1.29)*

*P < 0.05

## Discussion

This study assessed the FI-dementia association in middle-aged and older individuals during a 19-year follow-up in a large cohort of twins and analyzed whether familial effects (shared environment and genetics) affect the association. In the full sample, a 10% increase in the FI was associated with a 17% increase in the risk of dementia, after adjusting for age, sex, education, and tobacco use. In the cognitive sample, a 10% increase in FI associated with a 14% increase in the risk of dementia, after adjusting for age, sex, and baseline cognitive level. The FI-dementia association remained significant after further adjustment for *APOE* ɛ4 carrier status and when considering the competing risk of death. After controlling for familial factors in within-pair analyses and taking into account an age-varying (age at FI measurement) risk of frailty on dementia, we could neither find evidence that the effect sizes in DZ and MZ twin pairs significantly differed from the population-level estimates, nor that the MZ estimate significantly differed from the DZ estimate. These findings would suggest that familial factors, including genetics, do not account for the association between frailty and dementia. The risk carried by increased frailty on dementia was seemingly constant after age 50 until very old age. A sensitivity analysis using the FI-NTRF that was stripped from traditional dementia risk factors showed the association between frailty and dementia remained significant.

Our study adds to the current understanding of the relationship between frailty and dementia by showing that it is independent of familial factors, i.e., everything that makes the family members similar to each other, even in old age. This means that in identical twins, the twin with a higher level of frailty is more likely to develop dementia regardless of their similar genetic propensities and shared environmental risks, suggesting that the association between frailty and dementia is consistent with a causal hypothesis. Such study designs have not been used before to assess the relationship between frailty and dementia. Another novel contribution is the finding that the risk conferred by a higher level of frailty is similar in magnitude in midlife as in old age, suggesting that screening for frailty before or at midlife might provide benefit in identifying at-risk individuals. In the light of our previous finding that the rate of increase in frailty more than doubles after age 65 [32], extending clinical frailty assessments to younger age groups might indeed be beneficial in tackling frailty before it escalates and makes interventions less likely to succeed.

Meta-analyses and several observational studies [5, 33–35] have showed a significant association of frailty with the risk of dementia, independent of other risk factors and regardless of the scale used to measure frailty. Our study is in line with these findings as we observed that higher FI is associated with an increased risk of all-cause dementia after adjusting for age, sex, education, tobacco use, and cognition. Similar to Rogers et al. [34] who reported an association between higher FI and incident dementia in a competing risk model in the English Longitudinal Study of Aging, we found that the association remained significant after accounting for the competing risk of death. Regarding the role of the *APOE* genotype, our study is the second to find that the FI is associated with incident dementia independent of the *APOE* ε4 carrier status, consistent with Ward et al. [36] who recently found that the risk effect of the FI on incident dementia was similar in both carriers and non-carriers of the *APOE* ε4 allele. Lastly, similar to the findings by Rogers et al. [34], we found that the risk of frailty increased in a dose-response manner across the categorized FI (non-frail, pre-frail, and frail).

Although the association between frailty and dementia appears robust and seemingly independent of other risk factors, the role of familial environmental factors and genetic predisposition, other than *APOE*, in the association has not been studied. Using a unique twin design, our study assessed the impact of such environmental and genetic factors. Comparing the population-level estimates to the within-pair estimates in DZ twins in the full and cognitive samples, we did not find evidence of attenuation of the effect, indicating that shared environmental factors do not explain the frailty-dementia association. When further analyzing the difference between the DZ and MZ twin estimates in the within-pair analysis—that for the MZ twins fully adjusts for genetic factors—we found that although the DZ estimate appeared higher in the full sample and in the age-varying analysis, the difference was not statistically significant. Finally, when comparing the within-MZ twin estimate with the population estimate, the estimates were very similar and not statistically significantly different. This finding would lend support to the hypothesis that genetic factors do not explain the FI-dementia association either; note, though, that the within-pair analyses yielded estimates with wide confidence intervals, which indicates that inferences should be made with caution. Should further studies corroborate our findings that frailty predicts dementia independent of familial factors, frailty would be an actionable target for preventing or delaying dementia.

To the best of our knowledge, there are currently no trials aimed at preventing or delaying dementia through reversing or slowing down the progression of frailty. However, better management of frailty may contribute favorably to the prevention of dementia in several ways. Individuals living with frailty or at the risk of becoming frail can be subjected to medication reviews and fall prevention measures, decreasing the odds of escalating frailty and its negative sequelae that pave the way for dementia. The multidimensional nature of frailty nevertheless creates necessities for more comprehensive approaches, such as multicomponent interventions. A 6-month intervention based on group exercise, nutritional supplementation, depression management, deprescribing medications, and home hazard reduction showed sustained beneficial effects on frailty up to 1 year [37]. Moreover, as frailty can present both in the absence and presence of multimorbidity, disease management may be more beneficial to those living with comorbidities, while slowing down cellular aging might benefit those whose frailty is driven by accelerated biological aging rather than age-related diseases [38]. A geroscience hypothesis posits that targeting fundamental aging processes at the cellular level might delay the onset or severity of multiple chronic diseases and frailty [38, 39]. While still in its infancy, senolytic treatments to clear senescent cells and manipulation of signal transduction pathways linked to metabolism and nutrient sensing might be such geroscience approaches that have the potential to mitigate frailty [38, 39]. Another emerging approach for frailty therapeutics is drug repurposing in which existing drugs are used for new therapeutic targets. Our group has recently shown that lipid-lowering therapeutics might decrease the odds of frailty in midlife and older age [40]. Nevertheless, even if the above approaches showed beneficial effects on frailty, they are likely non-specific such that they affect the risk of dementia, too. As frailty and cognitive decline develop over a long period of time and share a common pathologic basis [9], finding approaches that specifically target frailty might be challenging. However, as we found that the relationship between the FI and the risk of dementia is linear, with even lower levels increasing the risk, targeting frailty long before dementia is manifest might provide a specific time window for future therapeutic approaches.

Our study also sheds light into the age-varying and sex-specific risk of frailty on dementia. In the within-pair analysis—that adjusts for covariates and familial factors—we found that the risk was seemingly constant from age 50 to 90. This finding indicates that even though the risk of dementia increases with age, the risk conferred by increased frailty is similar in magnitude from midlife into old age. In the sex-stratified analysis, the risks appeared similar in men and women, although in MZ men the effect size of FI decreased and attenuated to null—a finding that can be attributed to a small sample size or true null finding.

This study has several strengths. Firstly, we used a large, genetically informative sample of twins with a wide age range and a long follow-up to dementia (up to 19 years). Secondly, the role of familial factors in the frailty-dementia association has not been studied before, and our study provides a unique opportunity to do so. Thirdly, the BW model used in the within-pair analysis is a robust approach that typically produces a more powerful test of the within-pair effect than a stratified conditional Cox regression [22]. One of the limitations in our study is that the FI was measured based on self-reported items of which a large proportion were medical conditions, leading to potential misclassification and a FI that is skewed towards comorbidities. Some of the comorbidities are also known risk factors for dementia, potentially driving the association beyond the construct of frailty itself. Nevertheless, the estimates for FI-NTRF and FI-TRF were similar. In addition, pertinent to all aging studies, a relatively large number of individuals were censored due to death, leading to a limited number of informative MZ twin pairs, especially in the sex-stratified analysis. Furthermore, within-pair estimates, especially for MZ twins, were imprecise with wide confidence intervals, which warrants caution in conclusions based on the study. For example, the within-MZ pair estimate does not only cover the population estimate, it also covers the null (i.e., a HR of 1), and the results are thus compatible with the FI-dementia association being explained by the factors shared within MZ pairs (e.g., genetics). Nevertheless, the within-MZ pair estimate does not significantly differ from the population-level estimate. Lastly, for those dementia diagnoses that were obtained from the NPR, there is a possibility of uncertainty in the timing of the onset of dementia as the diagnoses in the NPR are recorded approximately 5 years after the age of onset [41].

## Conclusion

Our study confirms previous observational findings that increased frailty is associated with a higher risk of dementia. As a novel finding, the within-pair analysis supports the role of frailty as an independent, potentially causal risk factor for dementia across adulthood and into old age. Considering that frailty is a modifiable condition if identified early on, timely management of frailty might provide a target for decreasing or delaying the incidence of dementia.

## Supplementary Information

**Additional file 1: Appendix S1.** Supplementary methods. **Appendix S2.** Supplementary results. **Table S1.** The 44 frailty items and the coding rules**. Table S2.** ICD codes used to identify dementia. **Table S3.** ATC-codes for identification of dementia medication. **Table S4.** Descriptive statistics of the cognitive sample. **Table S5.** Descriptive statistics of the study population stratified by dementia diagnosis. **Table S6.** Descriptive statistics of the study population by sex. **Table S7.** Association of the frailty index (FI) with the risk of dementia using Cox regression in the cognitive sample (left panel) and in the genotyped samples II adjusting for the APOE ɛ4 carrier status (right panel). **Table S8.** Association of the frailty index (FI) with the risk of dementia using the competing risk model in the full (left panel) and cognitive (right panel) samples. **Table S9.** Association of the frailty categories with the risk of dementia using Cox regression in the full (left panel) and cognitive (right panel) samples**. Table S10.** Association of the frailty index (FI) with the risk of dementia in complete DZ and MZ twin pairs of the within-pair sample II in the multivariate Cox model (left panel) and in the within-pair model (right panel). **Table S11.** Sex-stratified associations of the frailty index (FI) with the risk of dementia within dizygotic (DZ) and monozygotic (MZ) twin pairs of the within-pair samples I and II using Cox regression**. Table S12.** Associations between the frailty index (FI) constructed from traditional (FI-TRF, Model 1) and non-traditional risk factors (FI-NTRF, Model 2) for dementia with the risk of dementia using Cox regression. **Figure S1.** The distribution of the frailty index (FI) in the full (A) and cognitive samples (B) by sex**. Figure S2.** The functional form of the association between the frailty index (FI) and the risk of dementia. **Figure S3**. Kaplan-Meier plots for the probability of being dementia-free during the follow-up by the frailty index categories. **Figure S4.** The ratio of the hazard ratios (HRs; HRDZ/HRMZ) in the within-pair interaction model. **Figure S5.** Hazard ratios (HRs) and the subdistribution HR (SHR) for the competing risk model and 95% confidence intervals (CIs) of incident dementia in relation to a 10% increase in the frailty 38 index (FI) in the cognitive sample.

## Data Availability

Data used in the current study are not publicly available. However, data are available upon request from the Swedish Twin Registry for researchers who meet the criteria for access to confidential data. Data from the SALT study are available from the Swedish Twin Registry steering committee (http://ki.se/en/research/the-swedish-twin-registry-1; contact: tvillingregistret@ki.se).

## Abbreviations

APOE: Apolipoprotein E
ATC: Anatomical Therapeutic Chemical
BDRS: Blessed Dementia Rating Scale
BMI: Body mass index
BW: Between-within
CDR: The Cause of Death Register
CI: Confidence interval
DZ: Dizygotic
FI: Frailty index
FI-NTRF: Frailty index constructed from non-traditional risk factors of dementia
FI-TRF: Frailty index constructed from traditional risk factors of dementia
HR: Hazard ratio
ICD: International Classification of Diseases
MZ: Monozygotic
NPR: National Patient Register
PDR: Prescription Drug Register
SALT: Screening Across the Lifespan Twin Study
TELE: Telephone cognitive screening instrument
