# Supplementary material for: Frailty and the risk of dementia: is the association explained by shared environmental and genetic factors?
Source: BMC Med. 2021 Oct 18;19:248. doi: 10.1186/s12916-021-02104-3 (PMC8522144; doi:10.1186/s12916-021-02104-3)
Supplement: Supplementary file 1 — Additional file 1: Appendix S1. Supplementary methods. Appendix S2. Supplementary results. Table S1. The 44 frailty items and the coding rules. Table S2. ICD codes used to identify dementia. Table S3. ATC-codes for identification of dementia medication. Table S4. Descriptive statistics of the cognitive sample. Table S5. Descriptive statistics of the study population stratified by dementia diagnosis. Table S6. Descriptive statistics of the study population by sex. Table S7. Association of the frailty index (FI) with the risk of dementia using Cox regression in the cognitive sample (left panel) and in the genotyped samples II adjusting for the APOE ɛ4 carrier status (right panel). Table S8. Association of the frailty index (FI) with the risk of dementia using the competing risk model in the full (left panel) and cognitive (right panel) samples. Table S9. Association of the frailty categories with the risk of dementia using Cox regression in the full (left panel) and cognitive (right panel) samples. Table S10. Association of the frailty index (FI) with the risk of dementia in complete DZ and MZ twin pairs of the within-pair sample II in the multivariate Cox model (left panel) and in the within-pair model (right panel). Table S11. Sex-stratified associations of the frailty index (FI) with the risk of dementia within dizygotic (DZ) and monozygotic (MZ) twin pairs of the within-pair samples I and II using Cox regression. Table S12. Associations between the frailty index (FI) constructed from traditional (FI-TRF, Model 1) and non-traditional risk factors (FI-NTRF, Model 2) for dementia with the risk of dementia using Cox regression. Figure S1. The distribution of the frailty index (FI) in the full (A) and cognitive samples (B) by sex. Figure S2. The functional form of the association between the frailty index (FI) and the risk of dementia. Figure S3. Kaplan-Meier plots for the probability of being dementia-free during the follow-up by the frailty index categories. Figure S [file 12916_2021_2104_MOESM1_ESM.pdf]

Additional file 1

Frailty and the risk of dementia: is the association explained by shared environmental and genetic factors?

Contents

Appendix S1. Supplementary methods 2

Cognitive impairment screening 2

Within-pair analysis using the between-within model. 2

Appendix S2. Supplementary results 3

Table S1. The 44 frailty items and the coding rules 4

Table S2. ICD codes used to identify dementia. 7

Table S3. ATC-codes for identification of dementia medication. 7

Table S4. Descriptive statistics of the cognitive sample.. 8

Table S5. Descriptive statistics of the study population stratified by dementia diagnosis. 9

Table S6. Descriptive statistics of the study population by sex. 10

Table S7. Association of the frailty index (FI) with the risk of dementia assessed by Cox regression in the cognitive sample (left panel) and in the genotyped samples II adjusted for *APOE ε4* carrier status (right panel) 11

Table S8. Association of the frailty index (FI) with the risk of dementia assessed by the competing risk model in the full (left panel) and cognitive (right panel) samples. 12

Table S9. Association of the frailty categories with the risk of dementia assessed by Cox regression in the full (left panel) and cognitive (right panel) samples 13

Table S10. Association of the frailty index (FI) with the risk of dementia in complete DZ and MZ twin pairs in the multivariate Cox model (left panel) and in the within-pair model (right panel). ... 14

Table S11. Sex-stratified associations between the frailty index (FI) and the risk of dementia in dizygotic (DZ) and monozygotic (MZ) twins 15

Table S12. The associations between dementia and the frailty index (FI) constructed from traditional risk factors for dementia (FI-TRF, Model 1) and non-traditional risk factors for dementia (FI-NTRF, Model 2) assessed by Cox regression in the cognitive sample 16

Figure S1. The distribution of the frailty index (FI) in the full (A) and cognitive samples (B) by sex. 17

Figure S2. Assessing the functional form of the association between the frailty index (FI) and the risk of dementia in the full sample. 18

Figure S3. Kaplan-Meier plots for being dementia-free during the follow-up for non-frail, pre-frail, and frail individuals in the full (A) and cognitive sample (B). 19

Figure S4. The ratio of the hazard ratios (HRs; HR_{DZ}/HR_{MZ}) in the within-pair interaction model in the within-pair sample I 20

Figure S5. Hazard ratios (HRs) and the subdistribution HR (SHR) for the competing risk model and 95% confidence intervals (CIs) of incident dementia in relation to a 10% increase in the frailty index (FI) in the cognitive sample.. 21

References 22

Appendix S1. Supplementary methods

Cognitive impairment screening

Those aged 65 and older in SALT received a telephone cognitive screening instrument (TELE) for baseline cognitive assessment. TELE includes a 10-item mental status questionnaire, 3-word recall, a word similarities task and questions about health and daily functioning (1). The TELE score ranges from 0 to 19. For those who performed poorly in the TELE assessment (less than 13.5 points), an informant was interviewed using the Blessed Dementia Rating Scale (BDRS) (2) to assess how much the individual's cognitive levels interfered with daily functioning. After combining the TELE and BDRS assessments, the cognitive status of each individual was classified into four levels as follows: 0 (cognitively intact), 1 (minor errors), 2 (performed poorly) and 3 (cognitive dysfunction) as previously described (1). Those classified as having cognitive dysfunction were excluded from the current analysis.

Within-pair analysis using the between-within model.

The between-within (BW) model (3) in twin pairs was applied in the framework of the Cox proportional hazards model with time since baseline measurement as the underlying timescale. The equation can be written as follows:

$$\lambda(t_{ij}) = \lambda_0(t_{ij})\tilde{Z}_i exp(\beta_B \overline{FI}_i + \beta_W FI_{ij} + \Sigma\delta_{B_k}\bar{C}_{i_k} + \Sigma\delta_{W_k}C_{ij_k})$$

where $\lambda(t_{ij})$ is the hazard function at time t_{ij} and $\lambda_0(t)$ is the baseline hazard. A shared random effect following a gamma distribution was included in the model (the cluster-specific frailty $\tilde{Z}_i$ was assumed to follow a gamma distribution). The main exposure, frailty index (FI) is split into a between-pair effect β_B that scales the contribution of the averaged value $\overline{FI}_i$ in the i-th twin pair, and a within-pair effect β_W that scales the observed value FI_{ij} for twin j in pair i. Adjustment for the other covariates was done similarly and is indicated in the equation via the averaged between-pair term $\overline{C}_{i_k}$ and the observed individual term C_{ij_k}, where k varies over education, tobacco use and cognitive function level. The effect of the FI in the model is modeled as a between-pair effect β_B capturing covariate effects shared by both members in the twin pair, and as a within-pair effect β_W capturing covariate effects specific to each individual and adjusted for shared effects (only the within-effects are tabulated in the results). The estimated $\exp(\beta_W)$ term adjusted for familial effects i.e., genetics and shared environmental effects is presented as a hazard ratio (HR) for the within-pair effect of the FI separately in DZ and MZ twin pairs (Table 4 and Figure 2). To

assess the age-varying HRs, the BW model was reformulated for both DZ and MZ twin pairs and written as follows:

$$\lambda(t_{ij}) = \lambda_0\left(t_{ij}\right)\tilde{Z}_i exp(\beta_B \overline{FI}_i + \beta_W FI_{ij} + FI_{ij} s_1(Age_{ij}; \beta_W) + \Sigma\delta_{B_k}\bar{C}_{i_k} + \Sigma\delta_{W_k} C_{ij_k})$$

where $s_1(t_{ij}; \beta_W)$ is a smooth function representing the within twin pair effect that varies over the age at FI assessment, and e β_W is a vector of parameters. The smooth parameters were modeled as natural splines with 3 degrees of freedom for the time-dependent HRs.

To assess the difference of the age-varying HRs in DZ and MZ twins, the DZ and MZ twins were analyzed in the same BW model with two interaction terms (FI and zygosity, FI, zygosity and age) introduced, and modeled model with the smooth function. The model is written as follows:

$$\lambda(t_{ij}) = \lambda_0\left(t_{ij}\right)\tilde{Z}_i \exp(\beta_B \overline{FI}_i + \beta_W FI_{ij} + FI_{ij} \cdot s_1\left(Age_{ij}; \beta_W\right) + \beta_1 zygosity + \beta_2 FI_{ij} \cdot zygosity + FI_{ij} \cdot zygosity \cdot s_1\left(Age_{ij}; \beta_2\right) + \Sigma\delta_{B_k}\bar{C}_{i_k} + \Sigma\delta_{W_k} C_{ij_k})$$

Here, $zygosity$ represents DZ or MZ twin, β_2 is the effect of the interaction term between FI and zygosity and Age_{ij} denotes age at FI measurement.

Appendix S2. Supplementary results

Of the 10,487 individuals (mean age =72.4, SD=5.9) in the cognitive sample, 2,355 were diagnosed with dementia, 4,786 died and 3,346 were censored during the follow-up (Table S4). The proportional hazards assumption was met for the cognitive sample.

In the cognitive sample, a 10% increase in the FI was associated with the risk of dementia, the estimate being HR 1.15 (95%CI 1.09, 1.20) without adjusting for cognitive level and HR 1.13 (95% CI 1.07, 1.18) after adjusting for cognitive level (all models adjusted for age, sex, education and tobacco use; Table S10, left panel).

Table S10 shows the associations of the FI with the risk of dementia in complete DZ and MZ twin pairs of the within-pair sample II in the multivariate Cox model (left panel) and in the within-pair model (right panel). Formal testing of the difference in the population level estimate and within-pair estimate (Table S10 right vs. left panel MZ estimate) indicated no significant difference (p=0.21) in the cognitive sample.

Table S12 shows the associations between dementia and FI constructed from traditional (FI-TRF, Model 1) and non-traditional risk factors (FI-NTRF, Model 2) for dementia with the risk of dementia using Cox regression in the cognitive sample adjusted for age, sex, education, tobacco

use and cognitive function level. The FI-NTRF was significantly associated with the risk of dementia (Table S12).

Table S1. The 44 frailty items and the coding rules

No.	Question	Coding	Dementia risk factor	
			Yes	No
1	How do you estimate your general health?	Excellent=0, Good=0.25, Average=0.5, Not so good=0.75, Bad=1		X
2	Do you think your health status prevents you from doing things you want to do?	Not at all=0, To some extent=0.5, A great deal=1		X
3	How many times a year do you get serious infections (other than respiratory)?	0-1 times=0, 2-4 times=0.5, 5 times or more=1		X
4	Do you have buzzing in the ears?	Both ears or one ear=1, No=0		X
5	Do you have or have you had angina pectoris	No=0, Yes=1		X
6	Do you have or have you had heart attack	No=0, Yes=1		X
7	Do you have or have you had heart failure	No=0, Yes=1		X
8	Do you have or have you had high blood pressure	No=0, Yes=1	X	
9	Do you have or have you had lipid disorder, for example high cholesterol or high triglycerides	No=0, Yes=1	X	
10	Do you have or have you had vascular spasm in the legs (intermittent claudication)	No=0, Yes=1		X
11	Do you have or have you had clot in the leg (venous thrombosis)	No=0, Yes=1		X
12	Do you have or have you had cerebral hemorrhage or clot in the brain (stroke)	No=0, Yes=1	X	

13	Do you have or have you had TIA attacks (temporary weakness or paralysis or reduction of sensibility)	No=0, Yes=1	X	
14	Do you have or have you had irregular cardiac rhythm/atrial fibrillation	No=0, Yes=1	X	
15	Do you have or have you had chronic lung disease (including chronic bronchitis and emphysema)	No=0, Yes=1		X
16	Do you have or have you had dizziness	No=0, Yes=1		X
17	Do you have or have you had rheumatoid arthritis	No=0, Yes=1		X
18	Do you have or have you had knee joint problem	No=0, Yes=1		X
19	Do you have or have you had sciatica	No=0, Yes=1		X
20	Do you have or have you had osteoporosis	No=0, Yes=1		X
21	Do you have or have you had hip joint problem	No=0, Yes=1		X
22	Do you have or have you had back pain	No=0, Yes=1		X
23	Do you have or have you had neck pain	No=0, Yes=1		X
24	Do you have or have you had diabetes (including old age diabetes, and excluding pregnancy diabetes)	No=0, Yes=1	X	
25	Do you have or have you had goiter	No=0, Yes=1		X
26	Do you have or have you had glandular diseases (excluding goiter)	No=0, Yes=1		X
27	Do you have or have you had gall bladder problem	No=0, Yes=1		X
28	Do you have or have you had liver disease (for example, cirrhosis)	No=0, Yes=1		X
29	Do you have or have you had gout	No=0, Yes=1		X

30	Do you have or have you had kidney disease	No=0, Yes=1	X	
31	Do you have or have you had stomach or intestine problems	No=0, Yes=1		X
32	Do you have or have you had recurring urinary tract problems	No=0, Yes=1		X
33	Do you have or have you had cancer, tumor disease or leukemia	No=0, Yes=1		X
34	Do you have or have you had migraine	No=0, Yes=1	X	
35	Do you have or have you had asthma	No=0, Yes=1		X
36	Do you have or have you had allergy	No=0, Yes=1		X
37	Do you have recurrent periods of coughing?	No=0, Yes=1		X
38	You felt depressed. Never, seldom, often or always during the past week?	Never or almost never=0, Seldom=0.5, Often, always or almost always=1	X	
39	You were happy. Never, seldom, often or always during the past week?	Never or almost never=1, Seldom=0.5, Often, always or almost always=0		X
40	You felt lonely. Never, seldom, often or always during the past week?	Never or almost never=0, Seldom=0.5, Often, always or almost always=1		X
41	Do you have or have you had any physical handicap	No=0, Yes=1		X
42	Do you have or have you had Crohn's disease or Ulcerative colitis	No=0, Yes=1		X
43	How is your vision?	Good=0, Reduced=0.5, Highly reduced or blind=1		X
44	How is your hearing?	Good=0, Reduced=0.5, Highly reduced=1	X	

112

Table S2. ICD codes used to identify dementia.

ICD-7	ICD-8	ICD-9*	ICD-10
(used before 1969)	(used 1969-1986)	(used 1987-1996)	(used 1997 and onwards)
**304** Senile psychosis	**290** Senile and presenile dementia	**290** Senile and presenile organic psychotic condition	**F00** Dementia in Alzheimer's disease
**305** Presenile psychosis	**293.0** Cerebral arteriosclerosis	**294B/ 294.1** Dementia in conditions classified elsewhere	**F01** Vascular dementia
**306** Psychosis with cerebral arteriosclerosis	**293.1** Other cerebrovascular disturbances	**331A/ 331.0** Alzheimer's disease	**F02** Dementia in other diseases classified elsewhere
		**331B/ 331.1** Pick's disease	**F03** Unspecified dementia
		**331C/ 331.2** Senile degeneration of brain	**F051** Delirium superimposed on dementia
		**331X/ 331.9** Cerebral degeneration, unspecified	**G30** Alzheimer's disease
			**G311** Senile degeneration of brain, not elsewhere classified
			**G318A** Other specified degenerative diseases of nervous system: Lewy body dementia

*In the Swedish adaptation of ICD-9, the 4th digit was replaced with a letter. While the National Patient Register used the Swedish version, the international ICD version was used in the Cause of Death Register (10), and both versions are therefore reported here.

Table S3. ATC-codes for identification of dementia medication.

**N06DA** Anticholinesterases
N06DA02 Donepezil<br>N06DA03 Rivastigmine<br>N06DA04 Galantamine<br>(N06DA01 Tacrine and N06DA05 Ipidacrine not prescribed in Sweden)
**N06DX** Other anti-dementia drugs
N06DX01 Memantine<br>(N06DX02 Ginkgo folium not prescribed in Sweden)

Table S4. Descriptive statistics of the cognitive sample. Data presented for the dizygotic (DZ) and monozygotic (MZ) twins include those individuals who were available for the within-pair analysis. Values are mean (standard deviation, SD) unless otherwise indicated.

	Cognitive sample	Within-pair sample II	
	N=10,487	DZ twin individuals N=4,376	MZ twin individuals N=1,547
Age at baseline	72.3 (5.9)	71.1 (5.1)	71.4 (5.4)
Age range at baseline	65-97	65-91	65-88
Women, N (%)	5,872 (56.0)	2,417 (55.2)	921 (59.5)
BMI	25.1 (3.5)	25.2 (3.5)	25.0 (3.6)
Tobacco user, N (%)	4,925 (47)	2,098 (47.9)	664 (42.9)
Years of education	8.9 (3.1)	9.1 (3.2)	9.3 (3.2)
Cognitive function score	16.2 (1.7)	16.4 (1.6)	16.5 (1.5)
§Physical activity, median (IQR)			
Born before 1926	1 (1)	1 (1)	1 (1)
Born after 1926	4 (1)	4 (1)	4 (1)
Living alone, N (%)	3,638 (34.7)	1,370 (31.3)	501 (32.4)
FI, median (IQR)	0.136 (0.125)	0.131 (0.119)	0.131 (0.114)
Categorized FI			
Non-frail, N (%)	2,635 (25.1)	1,189 (27.2)	398 (25.7)
Pre-frail, N (%)	6,407 (61.1)	2,653 (60.6)	966 (62.4)
Frail, N (%)	1,445 (13.8)	532 (12.2)	183 (11.8)
Dementia diagnosis during follow-up, N (%)	2,355 (22.5)	909 (20.8)	323 (20.9)
Time to diagnosis, median (IQR)	15.2 (8.3)	16.2 (7.2)	16.7 (6.4)
Died during follow-up, N (%)	6,513 (62.1)	1,646 (37.6)	582 (54.4)

Note. Participants who used tobacco products include current smokers, ex-smokers, and snuff users at baseline. Cognitive function score (ranging from 0 to 19 points) at baseline, a higher value means a better cognitive function. §Physical activity was assessed using a different questionnaire for those born before 1926 vs after 1926.

Abbreviations: BMI body mass index; DZ dizygotic; FI frailty index; IQR interquartile range; MZ monozygotic; N number

Table S5. Descriptive statistics of the study population stratified by dementia diagnosis. Values are mean (SD) unless otherwise indicated.

	Full sample (N=41,550)			Cognitive sample (N=10,487)		
	All	No dementia	Dementia	All	No dementia	Dementia
N of participants	41,550	38,367	3,183	10,487	8,132	2,355
Age at baseline	58.0 (10.1)	57.1 (9.7)	69.5 (8.3)	72.3 (5.9)	72.0 (5.9)	73.3 (5.6)
Women, N (%)	22,193 (53.4)	20,294 (52.9)	1,899 (59.7)	5,872 (56.0)	4,420 (54.4)	1,452 (61.7)
BMI	25.0 (3.5)	25.0 (3.5)	25.0 (3.4)	25.1 (3.5)	25.2 (3.6)	25.0 (3.4)
Tobacco users, N (%)	24,491 (58.9)	22,967 (59.9)	1,524 (47.9)	4,925 (47.0)	3,912 (48.1)	1,013 (43.0)
Years of education	10.6 (3.2)	10.7 (3.2)	9.0 (3.0)	8.9 (3.1)	9.0 (3.1)	8.6 (3.0)
Cognitive function score				16.2 (1.7)	16.4 (1.5)	15.6 (2.1)
§Physical activity, median (IQR)						
Born before 1926	1 (1)	1 (1)	1 (1)	1 (1)	1 (1)	1 (1)
Born after 1926	3 (2)	3 (2)	4 (1)	4 (1)	4 (1)	4 (1)
Living alone, N (%)	9,005 (21.7)	7,973 (20.8)	1,032 (32.5)	3,638 (34.7)	2,790 (34.3)	848 (36.1)
FI, median (IQR)	0.108 (0.108)	0.108 (0.114)	0.131 (0.119)	0.136 (0.125)	0.136 (0.125)	0.136 (0.125)
Time to follow-up, median (IQR)	16.0 (2.4)	16.0 (2.3)	15.8 (5.3)	15.2 (8.3)	15.4 (9.1)	14.9 (6.0)
Categorized FI						
Non-frail, N (%)	15,464 (37.2)	14,604 (38.1)	860 (27.0)	2,635 (25.1)	2,035 (25.0)	600 (25.5)
Pre-frail, N (%)	22,354 (53.8)	20,439 (53.3)	1,915 (60.2)	6,407 (61.1)	4,966 (61.1)	1,441 (61.2)
Frail, N (%)	3,732 (9.0)	3,324 (8.7)	408 (12.8)	1,445 (13.8)	1,131 (13.9)	314 (13.3)

Note. In the cognitive function score (ranging from 0 to 19 points), a higher value indicates better cognitive function. §Physical activity was assessed using a different questionnaire for those born before 1926 vs after 1926.

Abbreviations: BMI, body mass index; DZ, dizygotic; FI, frailty index; IQR, interquartile range; MZ, monozygotic; N, number; SD, standard deviation

Table S6. Descriptive statistics of the study population by sex. Values are mean (SD) unless otherwise indicated.

	Full sample (N=41,550)			Cognitive sample (N=10,487)		
	All	Men	Women	All	Men	Women
N of participants (%)	41,550	19,357 (46.6)	22,193 (53.4)	10,487	4,615	5,872
Age at baseline	58.0 (10.1)	57.6 (9.8)	58.4 (10.4)	72.3 (5.9)	71.9 (5.6)	72.7 (6.0)
BMI	25.0 (3.5)	25.6 (3.1)	24.5 (3.8)	25.1 (3.5)	25.3 (3.0)	25.0 (3.9)
Tobacco users, N (%)	24,491 (58.9)	12,345 (63.8)	12,146 (54.7)	4,925 (50)	2,854 (61.8)	2,071 (35.3)
Years of education	10.6 (3.2)	10.6 (3.2)	10.6 (3.2)	8.9 (3.1)	9.2 (3.3)	8.6 (2.9)
Cognitive function score				16.2 (1.7)	16.4 (1.7)	16.1 (1.7)
§Physical activity, median (IQR)						
Born before 1926	1 (1)	1 (1)	1 (0)	1 (1)	1 (1)	1 (0)
Born after 1926	3 (2)	3 (2)	4 (1)	4 (1)	4 (1)	4 (1)
Living alone, N (%)	9,005 (21.7)	3,410 (17.6)	5,595 (25.2)	3,638 (34.7)	965 (20.9)	2,673 (45.6)
FI, median (IQR)	0.108 (0.108)	0.097 (0.097)	0.119 (0.119)	0.136 (0.125)	0.119 (0.108)	0.148 (0.131)
Time to follow-up, median (IQR)	16.0 (2.4)	15.9 (2.41)	16.1 (2.40)	15.2 (8.3)	14.0 (9.3)	16.0 (7.3)
Categorized FI						
Non-frail, N (%)	15,464 (37.2)	8,142 (42.1)	7,322 (33.0)	2,610 (25.1)	1,297 (28.3)	1,313 (22.6)
Pre-frail, N (%)	22,354 (53.8)	10,117 (52.3)	12,237 (55.1)	6,351 (61.1)	2,844 (62.1)	3,507 (60.3)
Frail, N (%)	3,732 (9.0)	1,098 (5.7)	2,634 (11.9)	1,429 (13.8)	437 (9.6)	992 (17.1)
Dementia diagnosis during follow-up, N (%)	3,183 (7.7)	1,284 (6.6)	1,899 (8.6)	2,340 (22.5)	896 (19.6)	1,444 (24.9)

Note. In the cognitive function score (ranging from 0 to 19 points), a higher value indicates better cognitive function. §Physical activity was assessed using a different questionnaire for those born before 1926 vs after 1926.

Abbreviations: BMI, body mass index; DZ, dizygotic; FI, frailty index; IQR, interquartile range; MZ, monozygotic; N, number; SD, standard deviation

Table S7. Association of the frailty index (FI) with the risk of dementia assessed by Cox regression in the cognitive sample (left panel) and in the genotyped samples II adjusted for *APOE ε4* carrier status (right panel). Hazard ratios (HRs) and 95% confidence intervals (CIs) are presented for a 10% increase in the FI.

	**Multivariate Cox model**		**Multivariate Cox models adjusted for *APOE* ε4 carrier status**	
	Cognitive sample (N=10,487)		Genotyped sample II (N=3,156)	
	Model 1	Model 2	Model 1	Model 2
	HR (95% CI)	HR (95% CI)	HR (95% CI)	HR (95% CI)
FI	1.15 (1.09, 1.20)*	1.13 (1.07, 1.18)*	1.16 (1.05, 1.27)*	1.15 (1.04, 1.27)*
Age at FI measurement	1.14 (1.13, 1.15)*	1.13 (1.12, 1.14)*	1.13 (1.11, 1.15)*	1.14 (1.12, 1.16)*
Sex	0.86 (0.78, 0.94)*	0.84 (0.76, 0.92)*	0.81 (0.68, 0.97)*	0.80 (0.67, 0.96)*
Education years	0.97 (0.95, 0.98)*	0.99 (0.98, 1.01)	1.00 (0.98, 1.03)	1.00 (0.98, 1.03)
Tobacco use	1.17 (1.06, 1.28)*	1.20 (1.09, 1.31)*	1.14 (0.96, 1.35)	1.14 (0.96, 1.35)
Cognitive function score		0.79 (0.77, 0.81)*	0.88 (0.83, 0.92)*	0.88 (0.84, 0.93)*
APOE ε4 status (ref. non-carrier)				
Heterozygous (ε2/ε4 or ε3/ε4)				1.79 (1.50, 2.13)*
Homozygous (ε4/ε4)				5.04 (3.44, 7.38)*

Note. Model 1 in the right panel presents associations in the genotyped sample II without adjusting for *APOE* ε4 status, whereas Model 2 additionally adjusts for *APOE* ε4 status. *P<0.05

Table S8. Association of the frailty index (FI) with the risk of dementia assessed by the competing risk model in the full (left panel) and cognitive (right panel) samples. Subdistribution hazard ratios (SHRs) and 95% confidence intervals (CIs) from the competing risk model are presented for a 10% increase in FI.

	Full sample (N=41,550)		Cognitive sample (N=10,487)	
	Model 1	Model 2	Model 1	Model 2
	SHR (95% CI)	SHR (95% CI)	SHR (95% CI)	SHR (95% CI)
FI	1.54 (1.34, 1.76)*	1.52 (1.33, 1.75)*	1.43 (1.23, 1.67)*	1.41 (1.21, 1.64)*
Male sex	1.10 (1.03, 1.19)*	1.10 (1.02, 1.18)*	1.24 (1.14, 1.35)*	1.20 (1.10, 1.31)*
Age at FI measurement	1.10 (1.09, 1.10)*	1.10 (1.09, 1.10)*	1.03 (1.03, 1.04)*	1.02 (1.01, 1.03)*
Years of education		0.97 (0.96, 0.98)*	0.98 (0.97, 0.99)*	1.00 (0.99, 1.02)
Tobacco user		1.02 (0.94, 1.10)	0.94 (0.87, 1.03)	0.97 (0.89, 1.06)
Cognitive function score				0.78 (0.76, 0.80)*
Time varying effect of FI	0.97 (0.96, 0.98)*	0.97 (0.96, 0.98)*	0.97 (0.96, 0.98)*	0.97 (0.96, 0.98)*

Note. Time varying effect of FI represents the interaction term between FI and time since measurement; this term was introduced as it was statistically significant in the competing risk models in both full and cognitive samples. *P<0.05

Table S9. Association of the frailty categories with the risk of dementia assessed by Cox regression in the full (left panel) and cognitive (right panel) samples, adjusted for age, sex, education, tobacco use and cognitive function.

	Full sample (N=41,550)	Cognitive sample (N=10,487)
	HR (95% CI)	HR (95% CI)
Frailty (ref. non-frail)		
Pre-frail	1.19 (1.09, 1.29)*	1.14 (1.03, 1.26)*
Frail	1.54 (1.35, 1.75)*	1.39 (1.20, 1.62)*
Age	1.15 (1.14, 1.16)*	1.13 (1.12, 1.14)*
Male sex	0.88 (0.81, 0.95)*	0.85 (0.77, 0.93)*
Years of education	0.97 (0.96, 0.98)*	0.99 (0.98, 1.01)
Tobacco user	1.19 (1.10, 1.29)*	1.20 (1.09, 1.32)*
Cognitive function score		0.79 (0.77, 0.81)*

Note. Categorization of the frailty index was based on the frailty index as follows: non-frail (FI ≤0.08), pre-frail (FI>0.08-0.25) and frail (FI >0.25).*P<0.05

Table S10. Association of the frailty index (FI) with the risk of dementia in complete DZ and MZ twin pairs in the multivariate Cox model (left panel) and in the within-pair model (right panel). Hazard ratios (HRs) and 95% confidence intervals (CIs) are presented for a 10% increase in FI.

	Within-pair sample II			
	DZ twins N=2,176 pairs	MZ twins N=766 pairs	DZ twins N=2,176 pairs	MZ twins N=766 pairs
	**Multivariate Cox model**		**Within-pair model**	
	HR (95% CI)	HR (95% CI)	HR (95% CI)	HR (95% CI)
FI	1.19 (1.10, 1.29)*	1.05 (0.91, 1.19)	1.21 (1.07, 1.37)*	1.24 (0.94, 1.63)
Age at FI measurement	1.13 (1.11, 1.15)*	1.12 (1.09, 1.15)*	1.15 (1.13, 1.17)*	1.15 (1.11, 1.18)*
Sex	0.80 (0.69, 0.93)*	0.89 (0.68, 1.17)	0.70 (0.56, 0.87)*	0.92 (0.66, 1.28)
Education years	0.99 (0.97, 1.02)	0.97 (0.93, 1.01)	0.99 (0.95, 1.04)	0.94 (0.86, 1.04)
Tobacco use	1.16 (1.00, 1.35)	1.14 (0.88, 1.49)	1.27 (1.01, 1.62)*	0.75 (0.46, 1.23)
Cognitive function score	0.82 (0.78, 0.86)*	0.82 (0.76, 0.88)*	0.83 (0.78, 0.89)*	0.97 (0.86, 1.09)

Note. The left panel presents associations between the FI and dementia using multivariate Cox model, whereas the right panel presents the within-pair associations from the within-pair model. *P<0.05

Table S11. Sex-stratified associations between the frailty index (FI) and the risk of dementia in dizygotic (DZ) and monozygotic (MZ) twins assessed by Cox regression. The hazard ratios (HRs) and 95% confidence intervals (CIs) are presented for a 10% increase in the FI. All models were adjusted for age, education years, tobacco user and cognitive level score.

	**Women**		**Men**	
	**DZ twin pairs** HR (95% CI)	**MZ twin pairs** HR (95% CI)	**DZ twin pairs** HR (95% CI)	**MZ twin pairs** HR (95% CI)
**Within-pair sample I**				
Multivariate Cox model	1.23 (1.14, 1.33)*	1.15 (1.01, 1.31)*	1.22 (1.10, 1.35)*	1.07 (0.87, 1.31)
Within-pair model	1.20 (1.04, 1.38)*	1.24 (0.95, 1.63)	1.29 (1.08, 1.55)*	0.89 (0.61, 1.30)
**Within-pair sample II**				
Multivariate Cox model	1.20 (1.09, 1.32)*	1.08 (0.92, 1.28)	1.17 (1.02, 1.34)*	0.96 (0.75, 1.21)
Within-pair model	1.14 (0.97, 1.35)	1.33 (0.95, 1.85)	1.28 (1.01, 1.63)*	0.96 (0.58, 1.59)

*P<0.05

Table S12. The associations between dementia and the frailty index (FI) constructed from traditional risk factors for dementia (FI-TRF, Model 1) and non-traditional risk factors for dementia (FI-NTRF, Model 2) assessed by Cox regression in the cognitive sample adjusted for age, sex, education, tobacco use and cognitive function level.

	**Cognitive sample**	
	Model 1<br>HR (95% CI)	Model 2<br>HR (95% CI)
FI-TRF	1.11 (1.07, 1.15)*	
FI-NTRF		1.09 (1.04, 1.14)*
Male sex	0.85 (0.78, 0.94)*	0.84 (0.76, 0.93)*
Age at FI measurement	1.13 (1.12, 1.14)*	1.13 (1.12, 1.14)*
Years of education	0.99 (0.98, 1.01)	0.99 (0.98, 1.01)
Tobacco user	1.21 (1.10, 1.32)*	1.20 (1.09, 1.32)*
Cognitive function score	0.79 (0.77, 0.81)*	0.79 (0.77, 0.81)*

*P<0.05.

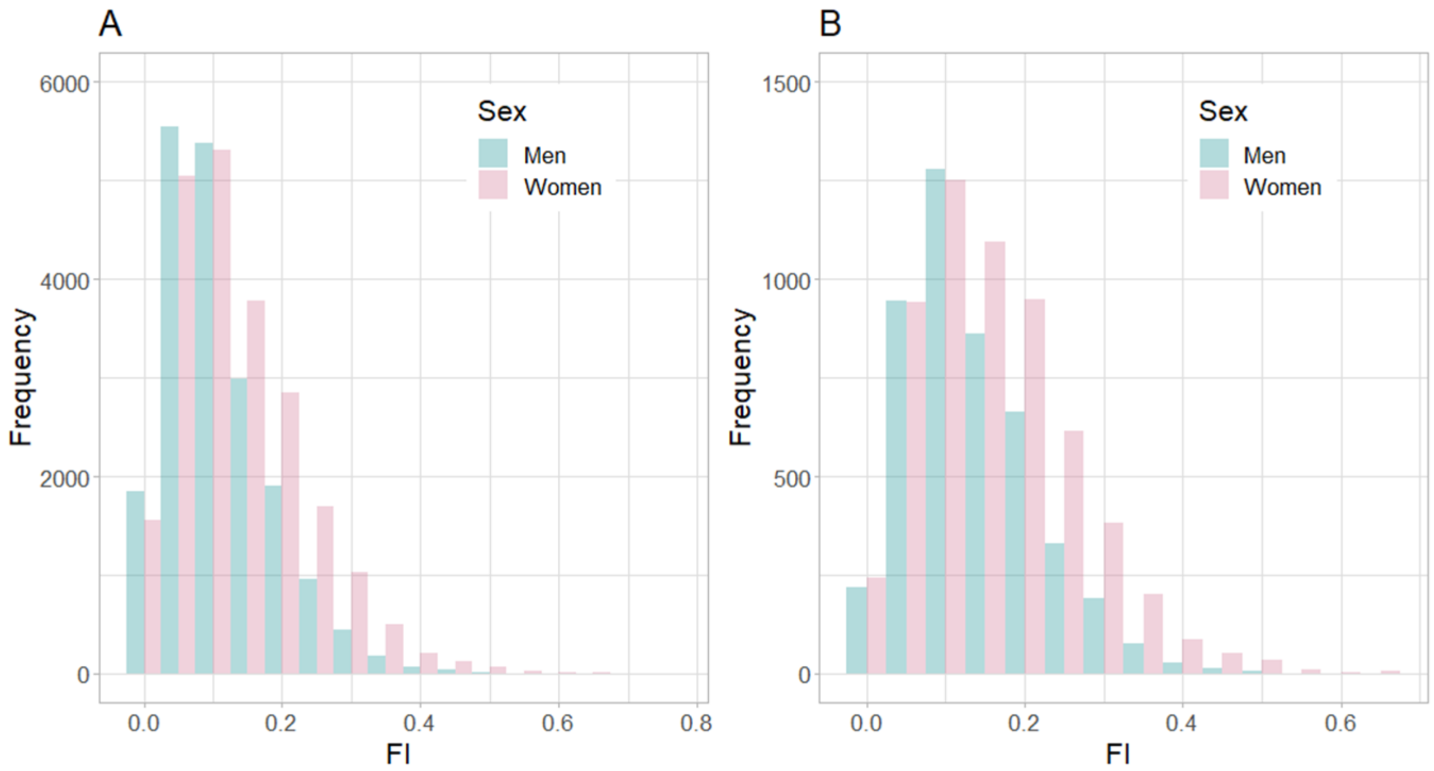

Figure S1. The distribution of the frailty index (FI) in the full (A) and cognitive samples (B) by sex.

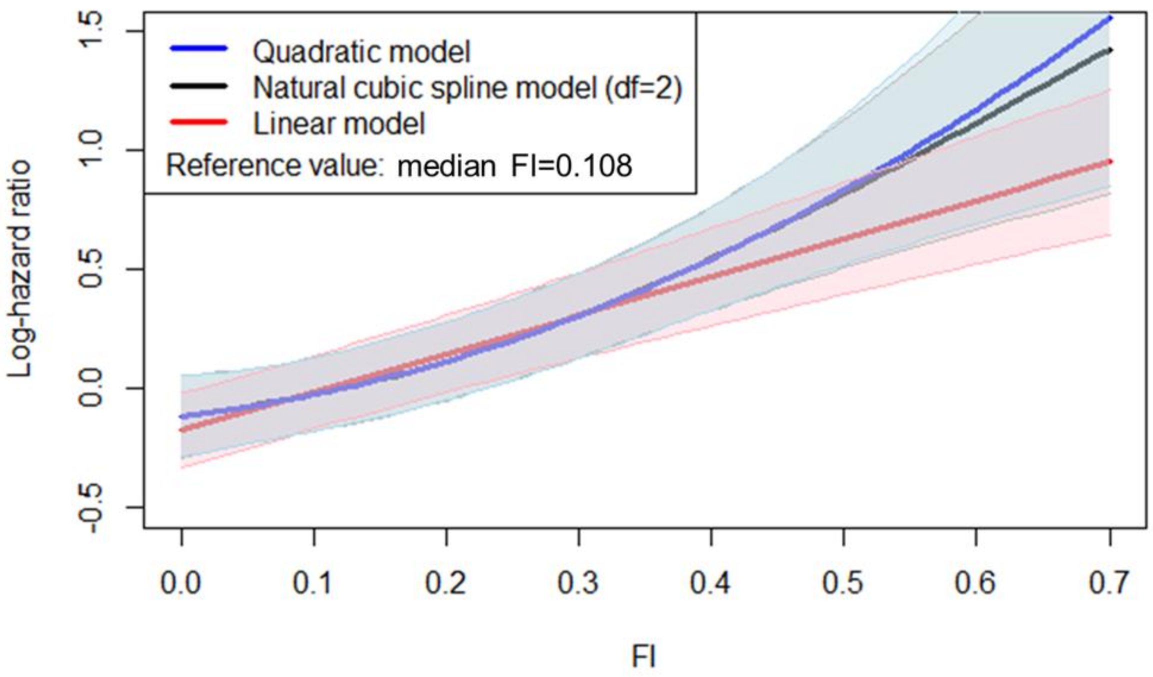

Figure S2. Assessing the functional form of the association between the frailty index (FI) and the risk of dementia in the full sample. The log-hazard ratios for the quadratic, cubic spline -transformed and linear FI are presented in reference to the cohort median FI=0.108. Abbreviations: df, degrees of freedom.

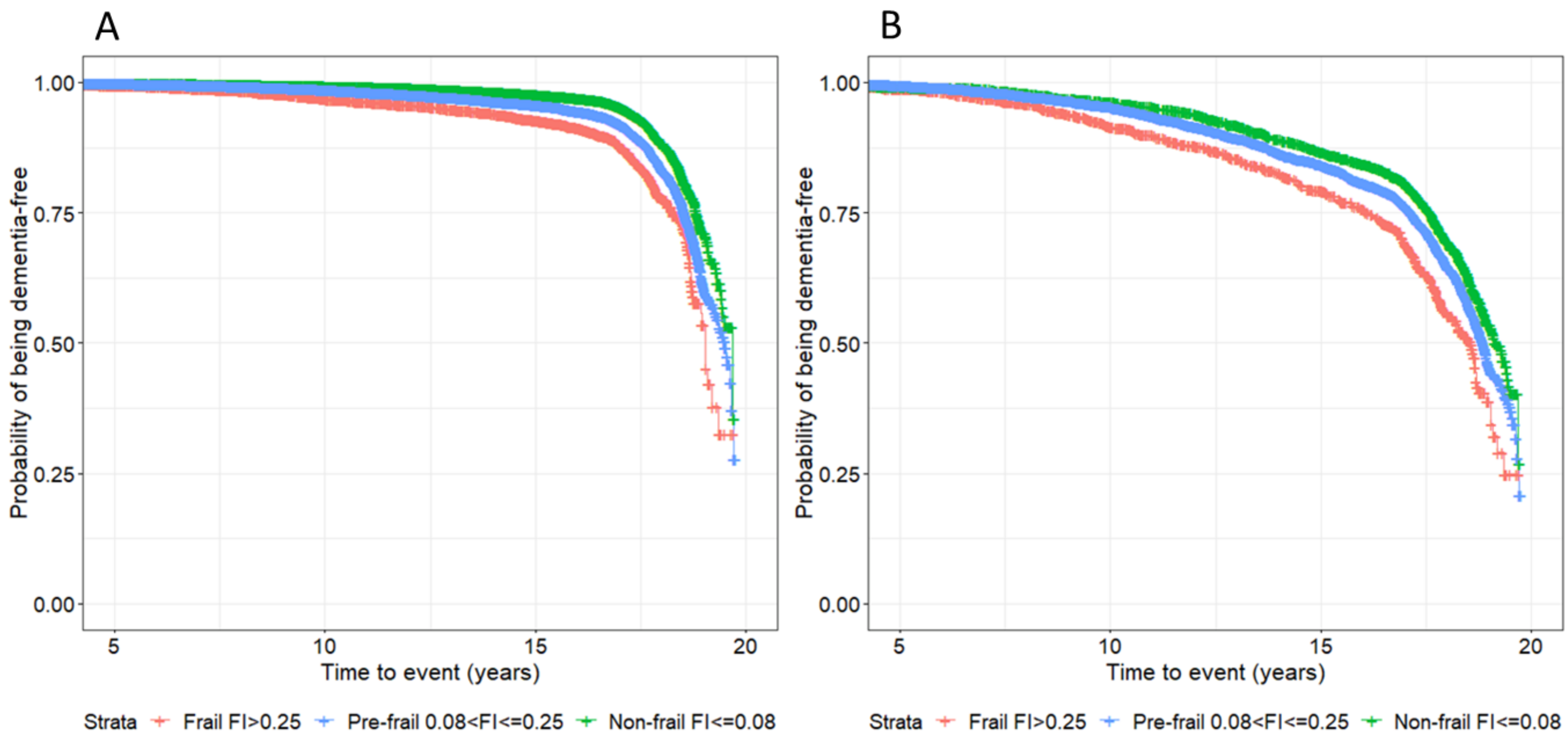

Figure S3. Kaplan-Meier plots for being dementia-free during the follow-up for non-frail, pre-frail, and frail individuals in the full (A) and cognitive sample (B).

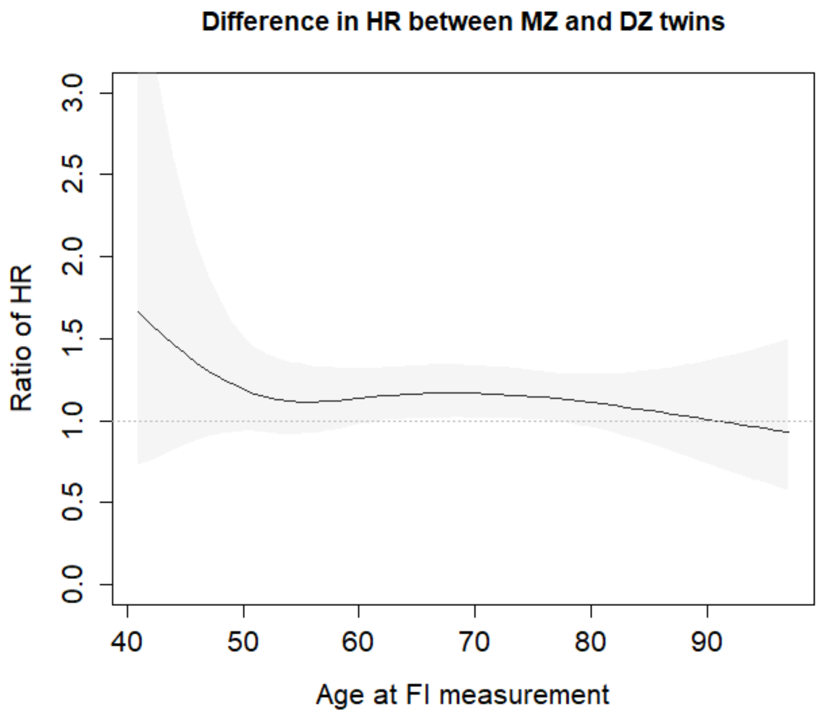

Figure S4. The ratio of the hazard ratios (HRs; HR_{DZ}/HR_{MZ}) in the within-pair interaction model in the within-pair sample I adjusted for age at frailty index measurement, sex, education years and tobacco use.

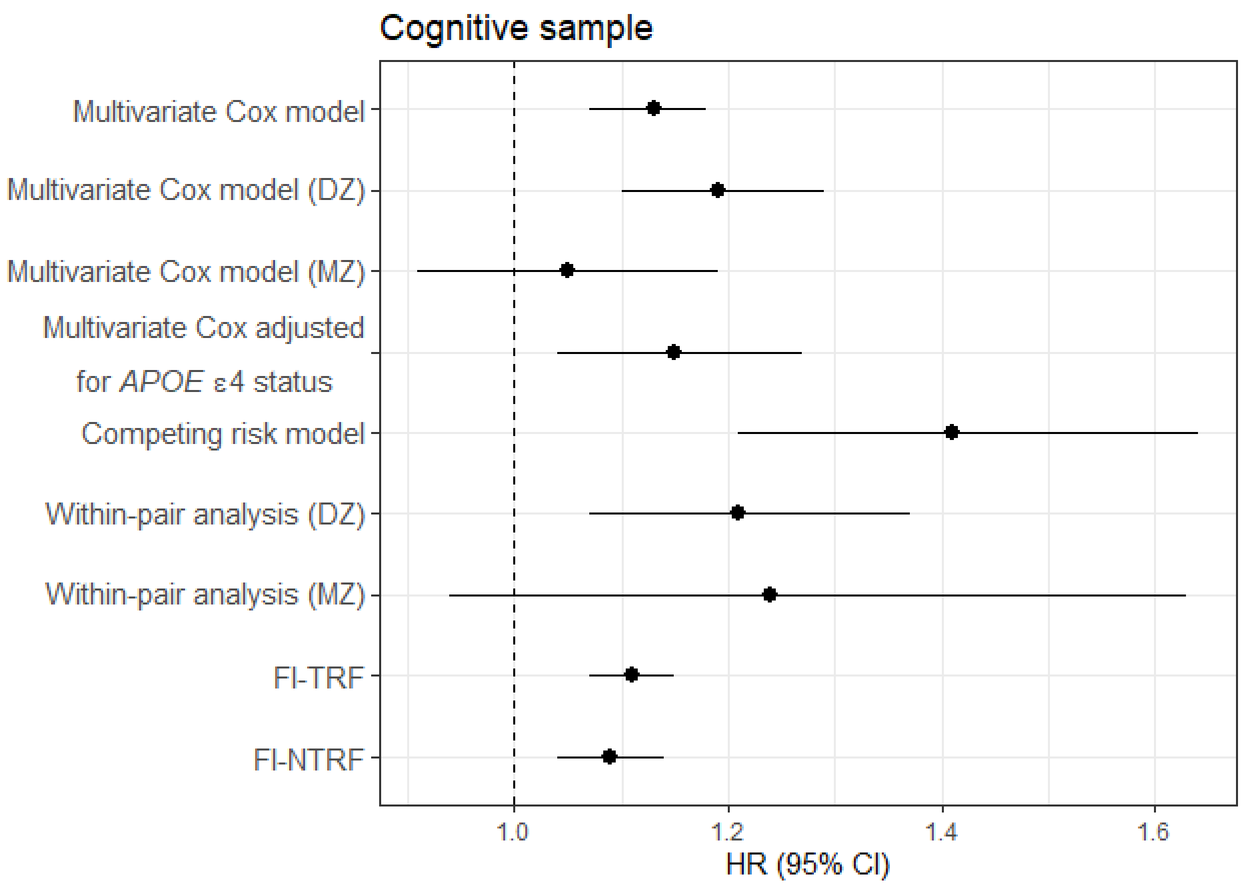

Figure S5. Hazard ratios (HRs) and the subdistribution HR (SHR) for the competing risk model and 95% confidence intervals (CIs) of incident dementia in relation to a 10% increase in the frailty index (FI) in the cognitive sample. Abbreviations: FI-NTRF, frailty index constructed from non-traditional dementia risk factors; FI-TRF; frailty index constructed from traditional dementia risk factors; DZ, dizygotic; MZ, monozygotic.

References

1. Gatz M, Fratiglioni L, Johansson B, Berg S, Mortimer JA, Reynolds CA, et al. Complete ascertainment of dementia in the Swedish Twin Registry: the HARMONY study. Neurobiol Aging. 2005;26(4):439-47.
2. Blessed G, Tomlinson BE, Roth M. The association between quantitative measures of dementia and of senile change in the cerebral grey matter of elderly subjects. Br J Psychiatry. 1968;114(512):797-811.
3. Sjölander A, Lichtenstein P, Larsson H, Pawitan Y. Between-within models for survival analysis. Statistics in medicine. 2013;32(18):3067-76.
